# Dissection of the DNA Mimicry of the Bacteriophage T7 Ocr Protein using Chemical Modification

**DOI:** 10.1016/j.jmb.2009.06.020

**Published:** 2009-08-21

**Authors:** Augoustinos S. Stephanou, Gareth A. Roberts, Laurie P. Cooper, David J. Clarke, Andrew R. Thomson, C. Logan MacKay, Margaret Nutley, Alan Cooper, David T.F. Dryden

**Affiliations:** 1 EastChem School of Chemistry, University of Edinburgh, Edinburgh EH9 3JJ, UK; 2 West Chem Department of Chemistry, University of Glasgow, Glasgow G12 8QQ, UK

**Keywords:** Ocr, overcome classical restriction, R/M, restriction/modification, EDC, 1-ethyl-3-(3-dimethylaminopropyl) carbodiimide hydrochloride, HOBt, hydroxybenzotriazole, MS, mass spectrometry, MALDI-TOF, matrix-assisted laser desorption/ionization time of flight, FT-ICR, Fourier transform ion cyclotron resonance, GdmCl, guanidinium hydrochloride, SAM, S-adenosyl-L-methionine, ITC, isothermal titration calorimetry, WT, wild type, DNA mimic, chemical modification, restriction/modification system

## Abstract

The homodimeric Ocr (*o*vercome *c*lassical *r*estriction) protein of bacteriophage T7 is a molecular mimic of double-stranded DNA and a highly effective competitive inhibitor of the bacterial type I restriction/modification system. The surface of Ocr is replete with acidic residues that mimic the phosphate backbone of DNA. In addition, Ocr also mimics the overall dimensions of a bent 24-bp DNA molecule. In this study, we attempted to delineate these two mechanisms of DNA mimicry by chemically modifying the negative charges on the Ocr surface. Our analysis reveals that removal of about 46% of the carboxylate groups per Ocr monomer results in an ∼ 50-fold reduction in binding affinity for a methyltransferase from a model type I restriction/modification system. The reduced affinity between Ocr with this degree of modification and the methyltransferase is comparable with the affinity of DNA for the methyltransferase. Additional modification to remove ∼ 86% of the carboxylate groups further reduces its binding affinity, although the modified Ocr still binds to the methyltransferase *via* a mechanism attributable to the shape mimicry of a bent DNA molecule. Our results show that the electrostatic mimicry of Ocr increases the binding affinity for its target enzyme by up to ∼ 800-fold.

## Introduction

**Fig. 1:**
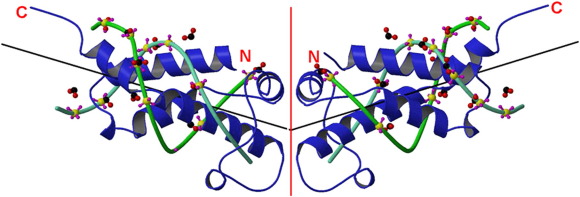
Superposition of two 12-bp B-DNA molecules on the ocr dimer.^5^ Ocr is shown in blue ribbon form, with the N- and C-termini indicated and the dimer interface shown as a red line. A fit of phosphate groups of a B-DNA complex onto 12 carboxyl groups of ocr gave an rms fit of 1.9 Å. Phosphate groups are shown in yellow (phosphorus) and purple (oxygen). The carboxyl groups are shown in red (oxygen) and black (carbon). The sugar backbones of the DNA chains are shown in two shades of green with the base pairs omitted for clarity. Vectors for the DNA helical axes are drawn as black lines.

The first protein to be produced during infection of *Escherichia coli* by bacteriophage T7 is *o*vercome *c*lassical *r*estriction (Ocr), the product of gene 0.3.^1^ The Ocr protein is the best characterised example of an antirestriction protein and of a structural mimic of DNA.^2–4^ Ocr is a highly negatively charged protein (p*I* of 4.02) with a shape similar to that of a bent double-stranded DNA molecule approximately 24 bp in length (Fig. 1).^5–13^ This molecular mimicry accounts for the ability of Ocr to inhibit, virtually irreversibly, all type I restriction/modification (R/M) enzymes found in the majority of eubacteria and archaea.^3,5,14^ Ocr binds to and completely occupies the DNA-binding site on the enzyme, thereby inhibiting the restriction endonuclease activity and protecting the phage genome as it enters the bacterium. Thus, Ocr greatly assists in the spread of phage infection in the bacterial population. The fact that Ocr mimics the general shape and charge of DNA, rather than any specific base pair sequence, means that the protein can act against all type I R/M enzymes, each of which recognises a different defined base pair sequence.^3,14^ The type I R/M enzymes are complex oligomeric multifunctional enzymes comprising an S subunit for DNA sequence recognition, two M subunits for DNA methyltransferase activity and two R subunits for ATP-hydrolysis-dependent DNA translocation and restriction endonuclease activities. The M_2_S_1_ complex can function as a sequence-specific methyltransferase, and the R_2_M_2_S_1_ complex can switch between methyltransferase and restriction endonuclease activities. The restriction endonuclease only operates on DNA containing unmethylated recognition sequences typically found on invading foreign phage DNA.

The structure of Ocr is an elongated, curved dimer. Each 116-amino-acid monomer is decorated with 34 surface-exposed Asp and Glu residues but only 2 Lys and 4 Arg residues.^5,6^ Asp and Glu residues are the only amino acids with side chains possessing multiple hydrogen-bond acceptors that are geometrically similar to oxygen atoms in a phosphate group. Many of the negatively charged residues of Ocr can be superimposed upon the equivalent phosphate groups on the DNA molecule recognised by the R/M enzyme. In addition to mimicking the charge distribution, Ocr mimics the bend of approximately 46° in the DNA helical axis induced in DNA when it binds to the R/M enzyme.^5^ The introduction of the bend in the DNA by the R/M enzyme is energetically costly. This cost is “saved” when the R/M enzyme binds to Ocr because Ocr is already “pre-bent.”^15^ By combining electrostatic mimicry of DNA and mimicry of the bent shape, binding of the R/M enzyme to Ocr is energetically more favourable than binding to DNA. Thus, the overall binding affinity of the R/M enzyme for Ocr is 50-fold greater than that for DNA with a *K*_A_ of ∼ 2 × 10^10^ M^− 1^.^8,9,11,13,16^

In this study, we explored the effect of chemically modifying the acidic residues in the Ocr molecule on its interaction with the archetypal EcoKI type I R/M system from *E. coli* K12 and assessed the relative importance of the electrostatic mimicry *versus* the mimicry of the bent shape of the DNA.

## Results

### Extent of modification

**Fig. 2:**
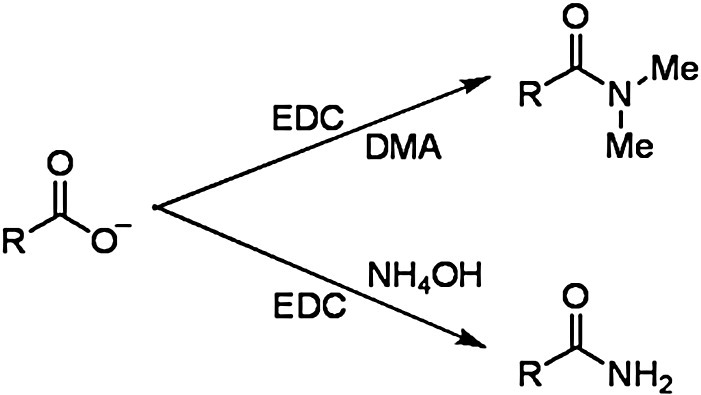
Derivatisation of carboxylate side chains of Ocr (Asp or Glu residues) using either dimethylamine (D-series) or ammonium hydroxide (N-series) as a nucleophile.

**Fig. 3:**
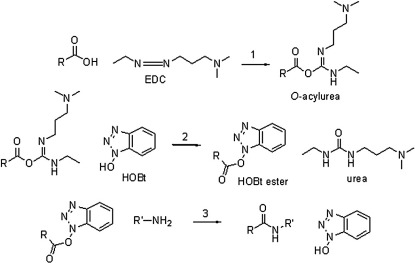
Reaction for the coupling of the carboxylate groups of the protein with an amine in the presence of EDC and HOBt.

We used 1-ethyl-3-(3-dimethylaminopropyl) carbodiimide hydrochloride (EDC), a water-soluble carbodiimide, to specifically modify the carboxyl groups of Asp and Glu residues and the C-terminus of Ocr (Fig. 2). The physical and chemical properties of EDC have been extensively studied.^17–20^ In aqueous solution under acidic conditions, EDC is anticipated to react with Asp, Glu, Cys and Tyr residues. The absence of a Cys residue in Ocr rules out any unwanted side reactions with sulfhydryl groups. Regeneration of unsubstituted tyrosyl residues, the phenolic hydroxyl group of which reacts with EDC to form a relatively stable *O*-arylisourea, is achieved by treating the protein with hydroxylamine.^21,22^ EDC reacts with surface-exposed carboxyl groups to form highly reactive *O*-acylisourea intermediates (Fig. 3, reaction 1) that are susceptible to nucleophilic attack. The reactions were done in the presence of a large molar excess of dimethylamine (i.e., D-series) or ammonium hydroxide (i.e., N-series) as a nucleophile. The end result is the formation of a stable amide bond with concomitant loss of one negative charge for each residue modified (Fig. 3). The *O*-acylisourea intermediate is however unstable and can either react with water, regenerating the initial carboxylate and hydrolysing EDC to its urea derivative, or rearrange to an *N*-acylurea, thus forming a stable adduct on the protein. This undesirable *N*-acylurea rearrangement is largely avoided by carrying out the reaction in the presence of (i) a high concentration of nucleophile and (ii) *N*-hydroxybenzotriazole (HOBt). HOBt reacts with the *O*-acylisourea to form a more stable activated ester (Fig. 3, reaction 2), greatly increasing the overall coupling efficiency (Fig. 3, reaction 3) and preferentially mediating the reaction with an amine.^23^ This can however include the ɛ- and α-amino groups of Lys residues and the protein N-terminus, respectively, leading to intramolecular cross-linking and possibly protein polymerisation. Nevertheless, HOBt minimises the adventitious formation of ester-bond cross-links because the HOBt ester is less susceptible than the *O*-acylisourea to nucleophilic attack by OH groups (i.e., Tyr, Ser and Thr residues). An additional mechanism has been reported by Nakajima and Ikada, whereby the *O*-acylisourea intermediate may react with a neighbouring free carboxylate to form an acid anhydride.^20^ This labile anhydride, which is highly susceptible to hydrolysis, can react with an amine and a hydroxyl to form an amide bond and an ester bond, respectively.^24^ It is probable that the modification reaction of surface-exposed carboxyl groups on Ocr occurs through a combination of the aforementioned species (*O*-acylisourea, HOBt ester and acid anhydride). Considering the close proximity of side chains within a protein and the variability of their p*K*_a_ values, depending on their specific microenvironment, it is clear that a limited number of side reactions are unavoidable.

**Fig. 4:**
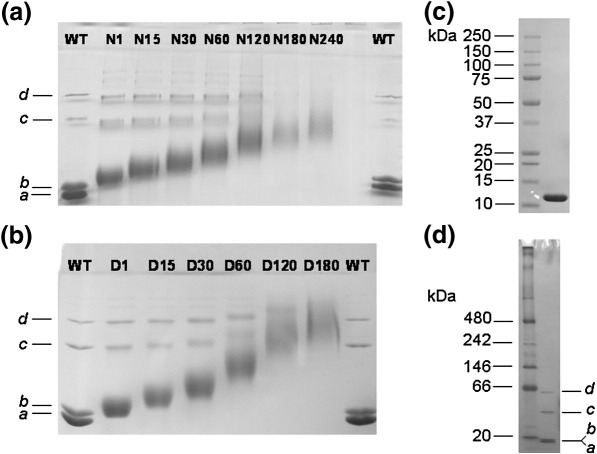
Gel electrophoretic analysis of chemically modified Ocr using either ammonium hydroxide (N-series) or dimethylamine (D-series) as a nucleophile. Panels (a) and (b) show native 15% PAGE of the N- and D-series of chemically modified Ocr samples, respectively. Gels were run under non-denaturing reducing conditions. “WT” refers to the WT unmodified Ocr. The name given to each modified protein sample reflects the reaction time in minutes. (c) SDS-PAGE, using NuPAGE 4%–12% Bis–Tris gel, of the unmodified Ocr sample [same sample as shown in lanes WT in panels (a) and (b)]. (d) Unmodified Ocr analysed by blue native gel electrophoresis using the NativePAGE Novex® 4%–16% Bis–Tris gel system. The protein markers were supplied by Invitrogen. The Ocr sample was resolved into four discrete bands labelled *a*–*d*.

In order to ascertain the degree of modification, we initially analysed the various protein samples by polyacrylamide gel electrophoresis (PAGE) under non-denaturing conditions (Fig. 4). The longer the modification reaction proceeded, the slower was the rate of migration through the gel, indicating progressively greater loss of negative charge. The modified samples gave protein bands that were more diffuse than the unmodified Ocr, which reflects the heterogeneous nature of the reaction products (Fig. 4a and b). As anticipated, the D-series of protein samples showed a greater change in migration rate since dimethylamine, being a better nucleophile, generated a greater level of modification than ammonium hydroxide after an equivalent reaction time. This is more apparent in the chromatographic analyses presented below.

Native gel electrophoresis of the unmodified Ocr revealed four sharp bands (Fig. 4a and b, bands *a*–*d*), even though the same sample analysed by denaturing SDS-PAGE migrated as a single species (Fig. 4c). We also analysed the unmodified Ocr on a NativePAGE Bis–Tris Gel System (Invitrogen) based on the blue native PAGE technique originally developed by Schägger and von Jagow.^25^ Blue native PAGE confirmed that bands *c* and *d* correspond to Ocr dimer and tetramer, respectively (Fig. 4d). As Ocr is stable in solution, we do not know precisely why the Ocr monomer (bands *a* and *b*) runs as two distinct bands, but this may be the result of partial denaturation during the running of the gel, thereby causing the protein to adopt a different conformation.

**Fig. 5:**
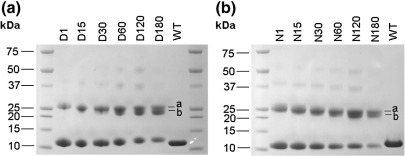
SDS-PAGE analysis of chemically modified Ocr using 15% acrylamide gel. (a) Analysis of the Ocr samples using dimethylamine as a nucleophile (D-series). The numbers for each sample correspond to the reaction time in minutes. (b) Analysis of the Ocr samples using ammonium hydroxide as a nucleophile (N-series). The numbers for each sample correspond to the reaction time in minutes. In both panels (a) and (b), the modified Ocr runs as two main bands: upper and lower bands corresponding to Ocr dimer and monomer, respectively. Note that the upper bands for the highly modified protein samples from the D- and N-series appear to resolve into two separate species (labelled *a* and *b*).

The chemically modified proteins were also analysed by SDS-PAGE, which confirmed the presence of cross-linked dimers (Fig. 5) most probably involving covalent-bond formation between Lys75 and a carboxylate on either side of the dimer interface. The formation of multimers by cross-linking of independent dimers was minimised by conducting the modification reaction at a low protein concentration (3 μM). For both the N- and D-series of chemical modifications, the percentage of cross-linked species increased with increasing reaction time. The upper band, corresponding to dimer, was noticeably broader and more diffuse than the lower monomer band. Indeed, for the highly modified samples, the upper band is resolved into two distinct species. This is probably caused by the incomplete unfolding of the more highly cross-linked dimers.

**Fig. 6:**
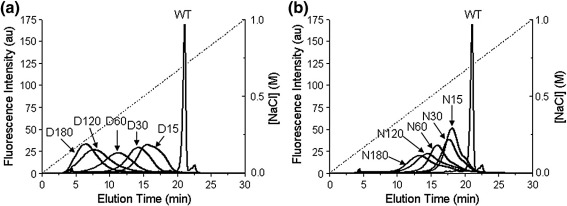
Anion-exchange chromatography profiles of Ocr and its chemically modified derivatives. Panels (a) and (b) show the analysis of the D- and N-series of chemically modified Ocr on a 1-ml Mono Q column, respectively.

Additional confirmation of chemical modification and loss of charge on the Ocr dimer was obtained by anion-exchange chromatography using a Mono Q anion-exchange column. Native Ocr binds very strongly to such media and requires high concentrations of NaCl to be eluted. In this experiment, Ocr eluted as a narrow peak late in the NaCl gradient (Fig. 6). The chemically modified samples eluted at progressively lower NaCl concentrations as the modification reaction time was increased. Moreover, the eluted material came off in a broad asymmetric peak, indicating considerable heterogeneity in each sample due to different numbers and locations of modified residues. Noteworthy is the asymmetric shape of the highly derivatised sample D180, the leading edge of which eluted from the column more abruptly than the other samples. We believe that the initial portion of the elution profile of D180 represents an endpoint in the chemical modification reaction in which almost all available Glu and Asp residues have been amidated.

Although the gel electrophoresis and ion-exchange experiments indicated that the protein was being derivatised, the number of modifications could not be quantified. Mass spectrometry (MS) of the D-series of modified samples was performed to obtain this information as each modification will increase the mass by 27 Da (Δmass + 27 Da). Unfortunately, MS is unsuitable for analysis of the N-series because there is only a net reduction in mass of 1 Da after each successive modification.

Matrix-assisted laser desorption/ionization time-of-flight (MALDI-TOF) MS of the D-series of modified samples was performed. Each spectrum showed a broad, slightly asymmetric peak skewed toward greater mass. Nevertheless, we were able to estimate the number of modifications for each sample as follows: D15, 9–18 modifications; D60, 16–25 modifications; D180, 22–33 modifications (data not shown). A smaller peak corresponding to Ocr dimer was also observed.

**Fig. 7:**
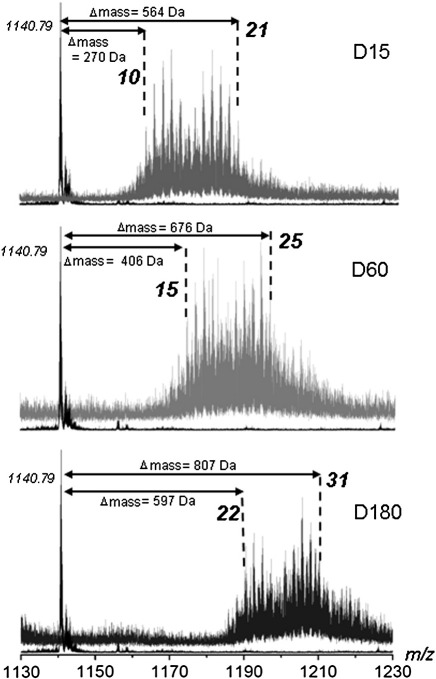
FT-ICR MS analysis of the D-series of chemically modified Ocr. Upper panel, Ocr before (left-hand peak) and after (nested set of peaks) chemical modification with dimethylamine for 15 min as described in Methods. Middle panel, Ocr before (left-hand peak) and after (nested set of peaks) chemical modification with dimethylamine for 60 min. Lower panel, Ocr before (left-hand peak) and after (nested set of peaks) chemical modification with dimethylamine for 180 min. In each case, the [M + 12H]^12+^ charge state was analysed. The number in italics is the average *m*/*z* value for unmodified Ocr. The number in bold signifies the number of Δ+27 Da modifications for each species.

D-series samples were also analysed on a Fourier transform ion cyclotron resonance (FT-ICR) instrument to obtain higher resolution. The proteins were desalted *via* reversed-phase HPLC, and electrospray MS data were collected for the peak eluting from the HPLC column. A nested set of MS species was observed with a mass difference of 27 Da, corresponding to the anticipated increase in mass after each modification with dimethylamine (Fig. 7). From these data, we were able to ascertain the number of amidations per Ocr monomer as being 10–21 modifications for D15, 15–25 modifications for D60 and 22–31 modifications for D180. Surprisingly, the nested set of peaks, corresponding to each successive modification, displayed a bimodal distribution that may arise from perturbations in the p*K*_a_ values of the carboxylates. This would influence reaction rates with the doubly protonated EDC molecule.

### Protein folding and stability

Modification of a large fraction of the 35 carboxyl groups (34 acidic residues plus the C-terminus) in each Ocr monomer could induce an alteration in the folding of the protein, potentially leading to a loss of activity through a gross structural defect rather than a more subtle change in the charge distribution of the protein. Chemical modification of Ocr did result in some loss of protein due to precipitation during sample preparation. However, upon removal of the aggregates, the final protein samples displayed no increased propensity to denature and precipitate, indicating that the structural integrity of the chemically modified Ocr was not compromised.

**Fig. 8:**
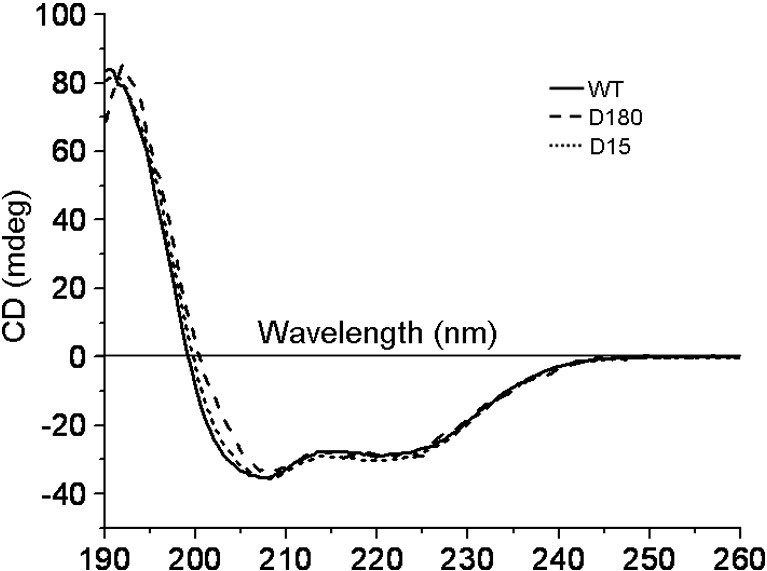
Far-UV CD spectroscopy of Ocr and chemically modified Ocr (D-series). There is no apparent change in the secondary structure of Ocr upon modification. CD analysis for the N-series of chemically modified Ocr samples gave similar results.

Chemically modified samples were subjected to CD spectroscopy in the far-UV region to detect the presence of protein secondary structure. The structure of Ocr is dominated by α-helices,^5^ and this is reflected in the CD spectra that display two distinct minima at 208 nm (part of the π-to-π⁎ transition) and 222 nm (*n*-to-π⁎ transition). CD spectra of the modified Ocr samples showed no major alteration in secondary structure content for the D-series of modifications (Fig. 8), nor for the N-series of modifications (data not shown). Spectra were analysed using the DichroWeb online secondary structure deconvolution programme.^26^ Analysis using the CDSSTR method gave an α-helical content of 60% for the unmodified Ocr, which is in good agreement with the crystal structure. Similar values were obtained for the chemically modified samples. We therefore concluded that the chemically modified proteins retained the overall fold of native Ocr.

We also investigated the stability of the chemically modified Ocr in comparison with that of the native protein. Initially, we analysed the guanidinium hydrochloride (GdmCl)-induced unfolding of the protein. Addition of denaturant caused the protein to unfold, resulting in a quenching of the fluorescent signal from the single tryptophan W94 (data not shown). For unmodified Ocr, the data indicated a two-state transition with no intermediate step with a Δ*G* of unfolding of 20 kcal mol^− 1^ and a midpoint of 3.85 M GdmCl, in good agreement with previously published data.^9^ However, the unfolding of the modified Ocr samples could not be described by a two-state transition. Instead, the midpoint of unfolding was calculated by fitting a sigmoidal curve (Origin Software version 6.1) to the data and found to occur at significantly higher concentrations of GdmCl for the N- and D-series of modified Ocr samples (D15, 4.25 M GdmCl; D60, 4.75 M GdmCl; N60, 4.88 M GdmCl; cf. unmodified Ocr, 3.85 M GdmCl). Thus, our data indicate that Ocr is actually stabilised by chemical modification.

The thermal stability of the N60 and D15 samples was also analysed by differential scanning calorimetry (data not shown). The data showed a clear energy uptake during the transition consistent with a cooperative endothermic unfolding event. The *T*_m_ of D15 (*T*_m_ = 68.2 °C) was similar to that of the unmodified Ocr (*T*_m_ = 69.0 °C). However, the *T*_m_ of N60 (*T*_m_ = 73.7 °C) was slightly elevated relative to that of the native protein. Noteworthy, however, was the observation that the unfolding of the modified Ocr samples (N60 and D15) was irreversible and showed a broader unfolding transition in contrast to unmodified Ocr, where the unfolding transition was completely reversible. This irreversible unfolding is presumably due to the formation of covalent cross-links within the Ocr dimer. Furthermore, a reduction in the net charge/electrostatic repulsions in the native fold could enhance the tendency for aggregation/irreversibility in the unfolded state.

### Interactions of modified Ocr with M.EcoKI and EcoKI

Having determined that the modified Ocr samples were still folded and the extent to which they had been modified, we assessed their ability to function as DNA mimics by measuring their interaction with the methyltransferase core, M.EcoKI, and the entire nuclease, EcoKI. Specifically, we performed (i) isothermal titration calorimetry (ITC) of Ocr with M.EcoKI to determine the enthalpy and stoichiometry of binding (the binding was too strong to give accurate values of the binding affinity), (ii) competition between Ocr and a fluorescently labelled 21-bp DNA duplex for binding to M.EcoKI to obtain the binding affinity and (iii) inhibition of the nuclease activity of EcoKI on plasmid DNA by Ocr.

The ITC experiment was initially performed using unmodified Ocr in 20 mM Tris–HCl, pH 8.0, 6 mM MgCl_2_, 7 mM 2-mercaptoethanol and 100 μM *S*-adenosyl-l-methionine (SAM) with or without 500 mM NaCl at a range of temperatures from 10 to 30 °C. The experiment at 25 °C was repeated using 20 mM Hepes buffer (heat of ionization = 20.5 kJ/mol) in place of Tris–HCl (heat of ionization = 47.4 kJ/mol) in the absence of NaCl, which gave a very similar enthalpy of interaction (Δ*H* of − 90.0 kJ/mol in Hepes compared with − 86.2 kJ/mol in Tris). These experiments indicated that there was no major contribution to Δ*H* due to effects of buffer ionization arising from protonation changes during binding. The enthalpy change upon interaction was strongly exothermic in the absence of NaCl but showed significant temperature dependence characteristic of a heat capacity change, ΔCp, upon formation of the M.EcoKI–Ocr complex. This was quantified from the slope of the plot of enthalpy change *versus* temperature (assumed to be linear) using the standard thermodynamic relationship:

ΔCp=dΔH/dT

**Fig. 9:**
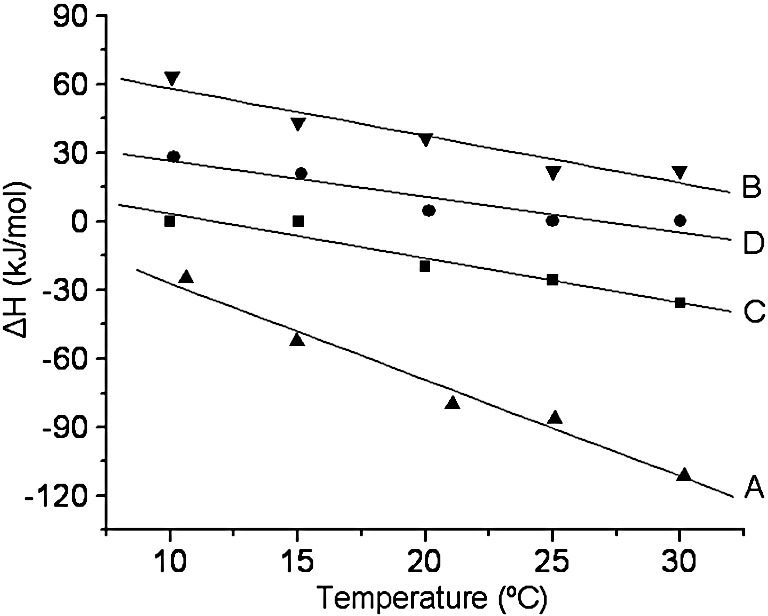
Plot of enthalpy, Δ*H* (kJ/mol), *versus* temperature (°C) for the interaction of either unmodified Ocr or chemically modified Ocr with M.EcoKI. The Δ*H* of interaction was determined from ITC experiments at temperatures ranging from 10 to 30 °C. “A” and “B” show unmodified Ocr in the absence and in the presence of 500 mM NaCl, respectively. “C” and “D” show the analysis of the interaction between M.EcoKI and the N60 and the D15 chemically modified Ocr samples in the absence of NaCl, respectively.

We determined the ΔCp of unmodified Ocr in low salt buffer to be − 4221 ± 372 J/mol K (Fig. 9). In 500 mM NaCl, the transition was less sharp (i.e., sigmoidal and indicating a slightly weaker interaction) and endothermic at all temperatures, and the slope of the linear regression yielded a binding heat capacity of − 2071 ± 372 J/mol K.

We also carried out ITC on a representative sample from the N- and D-series of chemically modified Ocr in the same zero NaCl buffer described above. Specifically, we chose the N60 and D15 samples because they eluted from the Mono Q column at a similar point in the NaCl gradient, suggesting that the Ocr was modified to a comparable extent. The calorimetric data showed that the interaction between chemically modified Ocr and M.EcoKI was stoichiometric for both N60- and D15-modified Ocr samples, as found for the unmodified Ocr (i.e., one Ocr dimer per molecule of M.EcoKI). In addition, the Δ*H* values fell between the behaviour of the unmodified Ocr in zero NaCl buffer and that in 500 mM NaCl buffer (Fig. 9). Furthermore, the ΔCp values for N60 Ocr (− 1385 ± 540 J/mol K) and D15 Ocr (− 1460 ± 385 J/mol K), although determined in the absence of NaCl, were close to the value determined for unmodified Ocr in 500 mM NaCl.

We also studied the interaction between modified Ocr and M.EcoKI using a sensitive fluorescence anisotropy assay to determine the binding affinity.^12,27^ The values of the dissociation constant, *K*_d_, given in Table 1 show that the modified Ocr samples were less able to interfere with DNA binding by M.EcoKI than the native Ocr protein. Furthermore, the amount of modification correlated with a loss in binding affinity. Specifically, the most extensively modified Ocr samples bound more weakly than DNA to M.EcoKI.

**Fig. 10:**
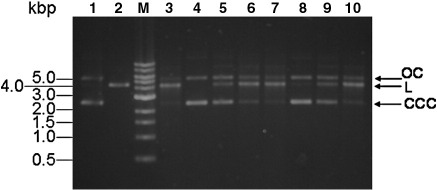
*In vitro* inhibition of EcoKI using chemically modified Ocr. An unmethylated plasmid (pBRsk1) with a single EcoKI recognition site was used as substrate. Samples were analysed using 0.9% agarose gel. Lane 1, undigested pBRsk1; lane 2, pBRsk1 linearised with EcoRI endonuclease to act as a size marker; lane 3, pBRsk1 linearised with EcoKI; lane 4, reaction performed using EcoKI pre-incubated with a 10-fold molar excess of unmodified Ocr dimer; lanes 5–10, as for lane 4 except the use of D15, D60, D120, N15, N60 and N120 in place of the unmodified Ocr, respectively. M, 1-kbp ladder from New England Biolabs. CCC is the covalently closed circular form of the plasmid, OC indicates the open circular form containing one or more single-strand nicks and L indicates the linear product of cleavage.

The activity of the modified Ocr samples was tested in an endonuclease assay using purified EcoKI. Linearisation of a circular unmethylated plasmid (pBRsk1) containing a unique EcoKI target recognition site was monitored in the absence and in the presence of Ocr (unmodified and N- or D-modified samples). In each case, the reaction mixture minus DNA was prepared and the digestion was initiated by addition of pBRsk1. The reaction was stopped after 10 min, and the mixtures were then analysed by agarose gel electrophoresis (Fig. 10). Incubation of pBRsk1 in the presence of a 6-fold excess of EcoKI resulted in complete cutting of the plasmid to a linear form within 10 min (compare lanes 1 and 3). The experiment was also performed in the presence of a 10-fold excess of Ocr dimer over EcoKI (lanes 4–10). As anticipated, unmodified Ocr completely abolished linearisation of the plasmid DNA (lane 4). However, the modified Ocr samples became progressively poorer endonuclease inhibitors as the extent of modification increased. For example, D15 and D60 displayed partial inhibitory activity and D120 showed almost no nuclease inhibition (lanes 5, 6 and 7, respectively). A similar trend was also found for the N-series of modified Ocr (N15, N60 and N120), although here the inhibitory activity was greater than that for the corresponding samples from the D-series (e.g., compare lanes 5 and 8).

## Discussion

The DNA mimicry displayed by Ocr comprises two main features: its mimicry of the bent DNA substrate, preferred by EcoKI, and its mimicry of the electrostatics of the phosphate backbone. Other features, such as H-bonding and van der Waals interactions, will also play a role in the binding of Ocr to its target enzyme. However, in the absence of a detailed structure for an Ocr–M.EcoKI complex, these intermolecular forces are not easily defined. Recently, Kennaway *et al*. produced a 16-Å resolution structure of M.EcoKI and an approximate atomic model of it in complex with Ocr, which shows it completely enveloping the Ocr molecule.^28^ Therefore, any of the residues of Ocr could potentially be involved in the interaction. Here, we focus on the electrostatic component of the Ocr–M.EcoKI interaction, which appears to be an important feature of the mimicry.

Chemical modification offers a convenient method of reducing the number of negatively charged groups (i.e., Asp and Glu side chains) on the Ocr surface. Our results clearly show the stepwise reduction of negative charge with reaction time. MS of the D-series of chemically modified samples showed that the protein was subject to averages of 16 (12/13/17/18/19 modifications most common), 20 (16/17/22/23/24 most common) and 27 (23/24/29/30 most common) modifications per Ocr monomer for the D15, D60 and D180 samples, respectively. The presence of cross-links was also demonstrated despite our effort to minimise this side reaction, so these numbers of modifications may be a slight underestimate by perhaps 1 or 2 modifications per Ocr monomer. Our MS measurements of the number of modified residues and the additional uncertainty due to cross-linking reflect the relative number of “random modification cycles” required to target the most important residues involved in the interaction. Therefore, our results are most probably an overestimate of the total number of negatively charged residues that are critically involved in the interaction between Ocr and M.EcoKI. This is supported by mutational studies showing that positional context and the number of negatively charged residues are critical for the interaction with M.EcoKI.^6,12,13,16^

The degree of chemical modification did not appear to have any deleterious effect on protein folding, as shown by CD, or stability. Such extensive modification without destruction of the protein fold is noteworthy. Indeed the stability to denaturation even increased. This is attributable to two factors. First, the adventitious formation of intermolecular (i.e., between monomers of an Ocr dimer) and intramolecular (i.e., within each Ocr monomer) cross-links during the chemical modification will stabilise the fold (although it will also make it recalcitrant to refolding upon denaturation). Second, the closeness of the carboxylates in the unmodified Ocr (approximately equal to the Bjerrum length) leads to electrostatic repulsion energies of the order *k*_B_*T*.^29^ Chemical modification increases the distance between the unmodified carboxylates, reducing electrostatic repulsion and enhancing stability.

Concomitant with the degree of modification, we observed a clear drop in the binding affinity for an anion-exchange column, for binding to M.EcoKI and for inhibiting the EcoKI nuclease. The first two features are more easily quantifiable and should be correlated given the importance of electrostatics in protein–DNA (and protein–DNA mimic) interactions. Additionally, the binding also gave rise to well-defined enthalpy and heat capacity changes, which can be discussed in terms of protein–protein interfaces.

**Fig. 11:**
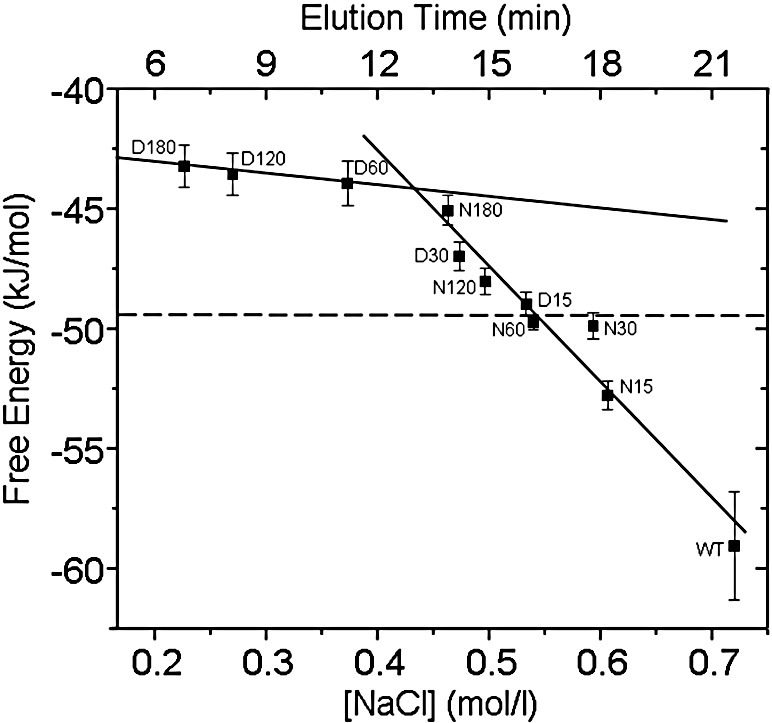
Correlation of the interaction between M.EcoKI and chemically modified Ocr samples with the NaCl concentration (mol/l) at which the Ocr sample elutes from an ion-exchange chromatography column (equivalent to the elution time from the column). The free energy of interaction calculated from the *K*_d_ values (Table 1) is plotted against [NaCl] (or elution time). Two regions showing a linear correlation of free energy of binding with [NaCl] are apparent. The dotted line represents the free energy of interaction between M.EcoKI and DNA.

Using our data of binding affinity and elution time (or [NaCl]) from the anion-exchange column, we can plot RTlog(*K*_d_) (i.e., the free energy) against [NaCl] or elution time (Fig. 11). Our results indicate that approximately 16 acidic residues in each Ocr subunit, equivalent to sample D15 if one ignores cross-linking modifications, must be lost for the protein to have the same binding affinity as DNA for M.EcoKI (i.e., 2.1 nM or − 49.5 kJ/mol). This is an ∼ 50-fold loss in binding affinity compared with unmodified Ocr. Further loss of acidic residues leads to further decrease in binding affinity. For example, sample D60, which has lost an average of 20 acidic residues, not including cross-linking modifications, displays an ∼ 500-fold loss in binding affinity relative to unmodified Ocr and binds more poorly than DNA. If one were to assume that each acidic residue of Ocr contributed equally to the interaction with M.EcoKI, one might anticipate a linear free energy relationship between log(*K*_d_) and the number of residues modified. As all degrees of modification from the wild type (WT; zero modifications) up to and including D30 and N180 fall on a straight line, this would appear to be the case starting from unmodified Ocr until one reaches ∼ 16 to ∼ 20 modified residues per monomer (∼ 32 to ∼ 40 for the dimer). Thus, each of these individual negative charges contributes only ∼ 0.4 to ∼ 0.5 kJ/mol to the free energy of binding. Since this energy is less than the thermal energy, *k*_B_*T*, then it is clear that a very large number of such weak interactions need to be summed to account for the effectiveness of Ocr as an inhibitor. This value per charge is also small when compared with the electrostatic effects observed with the conceptually similar barnase–barstar protein–protein interaction system.^30^ Additional modification further reduces binding affinity although the effect of individual single modifications is greatly reduced as the free energies of binding of samples D60, D120 and D180 are very similar. We can see that these highly modified bent Ocr molecules have a binding affinity of the order 20 nM (− 44 kJ/mol) for M.EcoKI. Thus, it appears that once one exceeds 16 to 20 modifications per ocr monomer, one observes only the contribution of the general shape of Ocr to the binding and the electrostatic mimicry is lost.

Hence, we have separated the relative contributions of the mimicry by Ocr, of the shape of the bent DNA molecule bound by EcoKI and of the charge distribution on the DNA molecule. It is noteworthy that the highly modified Ocr still displays significant binding (in the nanomolar range), presumably due to the favourable shape complementarity of the Ocr–M.EcoKI association involving a multiplicity of weak intermolecular interactions. Our results show that the addition of electrostatic mimicry to the DNA-shape mimicry of the Ocr molecule further increases the binding affinity for M.EcoKI by up to ∼ 800-fold.

We also investigated the Ocr–M.EcoKI interaction by ITC, and this gives us some further insight into the nature of the protein–protein interface. The thermodynamics of protein–protein interactions are typically made up of numerous small changes in free energy with both enthalpic and entropic components.^31^ One might anticipate a major entropic component to the interaction given that the interface between the two proteins is substantial, with the M.EcoKI almost completely engulfing the Ocr protein, which has a Connolly surface of 11,238 Ǻ^2^ for the dimer.^5,11^ A recent model for the Ocr–M.EcoKI interaction that is consistent with this hypothesis has been proposed.^28^ A large number of water molecules with all of their H-bonding potential are presumably displaced during the formation of the complex, and the dynamics of residues on the interface must become rather constrained. This is particularly important under high salt conditions where the counterions decorating the surface of Ocr must be displaced in order to allow interaction with M.EcoKI. Indeed, the transition in the presence of salt is more endothermic than that in the absence of salt at any given temperature (Fig. 9).

Initially, we studied the interaction of unmodified Ocr with M.EcoKI in the absence and in the presence of monovalent salt. Bearing in mind the average ΔCp for protein–protein interactions is reported to be − 1393 ± 845 J/mol K,^32^ our results show a surprisingly large ΔCp effect for the M.EcoKI–Ocr interaction that is strongly dependent on ionic strength. Similarly, we determined the ΔCp values of the D15 and N60 protein samples under low salt conditions, which were found to be similar to the value determined for unmodified Ocr under high salt conditions. Large ΔCp values similar to those obtained here have been found for the interaction between the highly charged inhibitor protein barstar and its target barnase enzyme.^30^

Conventionally, large ΔCp effects are associated with hydrophobic interactions. However, theoretical considerations and empirical observations show that long-range electrostatic interactions and other effects can also make a significant contribution to ΔCp.^30,33,34^ Our results clearly reinforce this notion. Furthermore, several algorithms that attempt to correlate the magnitude of ΔCp with changes in the solvent-accessible surface area of interaction have been devised.^35^ Assuming that the structure of the Ocr–M.EcoKI interface is essentially unchanged by high ionic strength, or by chemical modification, then conventional theories would predict the same ΔCp. Our results clearly contradict this prediction. Similar discrepancies with this surface area theory have been observed for the strong electrostatic interaction between the enzyme barnase and its protein inhibitor barstar.^30^

In conclusion, our results show that the DNA mimicry displayed by Ocr is extremely robust and can be separated into mimicry of the shape and charge of DNA. After extensive modification, potentially removing ∼ 86% of the negative charge from the carboxyl side chains and C-terminus, the modified Ocr protein still binds to M.EcoKI with an affinity of 27 nM (− 43.9 kJ/mol). This is only marginally weaker than the M.EcoKI–DNA interaction (− 49.4 kJ/mol) and is presumably the contribution to binding of M.EcoKI by Ocr's mimicry of the shape of the bent DNA molecule preferred by M.EcoKI.^5,15^ The introduction of negative charge onto this simple “shape mimic” further increases binding affinity for M.EcoKI until Ocr far surpasses the affinity of DNA for M.EcoKI. The introduction of the mimicry of the charge pattern on the DNA increases affinity to 44 pM (a further − 14.6 kJ/mol in addition to the shape mimicry). Thus, although the charge mimicry makes a lesser contribution than shape mimicry to the effectiveness of Ocr, it is a crucial addition to push the affinity of Ocr for M.EcoKI past the affinity of DNA for M.EcoKI, thereby transforming Ocr into an extremely effective inhibitor of type I R/M enzymes.

## Methods

### Enzymes, chemicals and plasmids

EcoKI (R_2_M_2_S_1_) and the DNA methyltransferase component M.EcoKI (M_2_S_1_) were purified as described previously.^36,37^ The Ocr protein was purified as described previously.^12^ Plasmid pAR2993 (a kind gift from Alan Rosenberg and William Studier, Brookhaven National Laboratory) harbours the gene encoding WT Ocr located just downstream of an isopropyl-β,d-thiogalactopyranoside-inducible promoter.^9^ pBRsk1 is an engineered version of pBR322 (4361 bp) in which one of the two EcoKI sites (4024–4036) has been removed by site-directed mutagenesis.^38^ The unmethylated form of pBRsk1 used in the nuclease assays was prepared from *E. coli* NM1261 (r^− ^m^− ^) (a kind gift of Prof. Noreen E. Murray, University of Edinburgh). Dimethylamine, EDC and HOBt were obtained from Pierce (Rockford, IL). Guanidine hydrochloride (ULTROL grade) was purchased from Calbiochem (San Diego, CA). SAM was from New England Biolabs (Ipswich, MA). All other reagents were purchased from Sigma-Aldrich (St. Louis, MO). Broad-range pre-stained molecular mass markers for SDS-PAGE were purchased from BioRad (Precision Plus Protein Standards; Hercules, CA). All solutions were made up in distilled, deionized water.

### Chemical modification procedure

Surface carboxyl groups of Ocr were chemically modified using EDC and either ammonium hydroxide (to give the so-called N-series of chemically modified proteins) or dimethylamine (to give the so-called D-series) as a nucleophile. Chemical modification of Ocr (3 μM) was carried out at 25 °C in 750 mM ammonium hydroxide or dimethylamine HCl, 150 mM NaCl, 60 mM EDC and 60 mM HOBt, pH 6.5. Aliquots were withdrawn at specific time points (1 to 180 min), and the reaction was quenched by adding a 6-fold excess of sodium acetate (from a 3 M stock of sodium acetate, pH 6.5). After incubation for 10–20 min, the mixture was first dialysed against 100 mM nucleophile (either ammonium hydroxide or dimethylamine, pH 8.5) for 4 h at 25 °C and then against 50 mM ammonium acetate at 4 °C for a minimum of 4 h. Hydroxylamine was added to the solution to a final concentration of 400 mM (from a 3 M stock of hydroxylamine, pH 7.0), and the mixture was incubated at 25 °C for 4 h. Finally, the solution was dialysed against 20 mM ammonium acetate and then the protein concentration was adjusted to 20–30 μM using a Vivaspin concentrator (10,000 MWCO; VivaScience AG). Samples were stored in 50% v/v glycerol at − 20 °C.

### Ion-exchange chromatography

WT and chemically modified Ocr samples were analysed by anion-exchange chromatography using a 1-ml Mono Q column (GE Healthcare). Each protein (11.2 μg) was individually loaded onto the column pre-equilibrated in 20 mM Tris–HCl, pH 8.0, at a flow rate of 1 ml/min. After washing the column, a linear gradient of 0–1 M NaCl in 20 mM Tris–HCl, pH 8.0, was run over 30 min using the same flow rate. Protein elution from the column was monitored by measuring the tryptophan fluorescence (excitation, 295 nm; emission, 350 nm) of the eluate. The elution time of each sample was determined by integrating the peak and calculating the point corresponding to 50% of the peak area (i.e., time at which 50% of the protein has eluted from the column).

### MALDI-TOF MS analysis

MS of the D-series of chemically modified Ocr was performed by MALDI-TOF using a Voyager DE STR instrument (Applied Biosystems, Foster City, CA). Protein samples were diluted in 0.1% trifluoroacetic acid to 0.05 mg/ml and mixed with an equal volume of matrix (saturated solution of sinapinic acid in 50% acetonitrile and 0.1% trifluoroacetic acid) on a stainless steel surface. The samples were air dried at room temperature to crystallize. The machine was operated in positive ion mode and calibrated with conalbumin and bovine serum albumin.

### High-resolution LC-MS analysis

Protein samples were extensively desalted by dialysis into 20 mM ammonium acetate prior to MS. For LC-MS, an Ultimate 3000 HPLC system (Dionex Corporation, Sunnyvale, CA) equipped with a monolithic PS-DVB (500 μm × 5 mm) analytical column (Dionex Corporation) was used. Solutions B and C were prepared comprising 2:97.95:0.05 and 80:19.95:0.05 of acetonitrile/water/formic acid, respectively. Samples in solution B containing ∼ 1 μg of chemically modified Ocr were centrifuged (16,100***g*** for 2 min) immediately prior to injection onto the column. After injection, the column was washed with solution B for 5 min, followed by a 20-min linear gradient elution (20 μl/min) into solution C. The eluate was passed into the mass spectrometer. MS data were acquired on a Bruker 12-Tesla Apex Qe FT-ICR (Bruker Daltonics, Billerica, MA) equipped with an electrospray ionization source. Desolvated ions were transmitted to a 6-cm Infinity Cell^®^ Penning trap. Trapped ions were excited (frequency chirp of 48–500 kHz at 100 steps of 25 μs) and detected between *m*/*z* values of 600 and 2000 for 0.5 s to yield broadband 512-kWord time-domain data. Fast FTs and subsequent analyses were performed using DataAnalysis (Bruker Daltonics) software. Multiple charge states could be observed in this way for each of the major species.

### Circular dichroism (CD) analysis

CD measurements were performed as described previously.^39^ Protein samples were prepared in 10 mM Tris–HCl, pH 8.0, and 50 mM NaF. Spectra (190–260 nm) were obtained at a protein concentration of 30 μM using a 0.2-mm path-length cell. Each spectrum was an accumulation of four individual scans. The spectra were corrected for buffer contribution.

### Unfolding studies

Equilibrium unfolding of Ocr as a function of GdmCl concentration was monitored by tryptophan fluorescence spectroscopy as described previously.^9^ The fluorescent intensity was measured as a ratio of the fluorescent signal at 350 *versus* 380 nm to remove any variation in intensity due to slight differences in protein concentration between samples.^36^

### Calorimetry

Differential scanning calorimetry and ITC were carried out as described previously.^12^ All ITC experiments were conducted using 20 mM Tris–HCl, pH 8.0, 6 mM MgCl_2_, 7 mM 2-mercaptoethanol and 100 μM SAM. Typically, Ocr at a concentration of 40 μM was titrated into an M.EcoKI solution at a concentration of 4 μM.

### Fluorescence anisotropy

Competition for binding WT or chemically modified Ocr to M.EcoKI was determined using the fluorescence anisotropy assay as described previously.^12^

### Nuclease assay

The *in vitro* assay monitored the cleavage of unmethylated circular pBRsk1 using purified EcoKI in the absence or in the presence of WT or chemically modified Ocr essentially as described elsewhere.^39^

## Figures and Tables

**Table 1:** *K*_d_ values for N- and D-series samples binding M.EcoKI as determined by fluorescence anisotropy (cf. *K*_d_ for unmodified Ocr is ∼ 44 pM, and *K*_d_ for DNA is ∼ 2.1 ± 0.35 nM)

	*K*_d_ (nM)
*D-series*	
D15	2.64 ± 0.52
D30	5.8 ± 1.4
D60	19.7 ± 7.3
D120	23.1 ± 8.1
D180	26.6 ± 9.2

*N-series*	
N15	0.56 ± 0.13
N30	1.82 ± 0.37
N60	1.9 ± 0.2
N120	3.77 ± 0.82
N180	12.6 ± 3.0

M.EcoKI was titrated into a solution containing 50 nM Ocr and 2 nM concentration of a 21-bp DNA duplex fluorescently labelled at the 5′ end. The binding of M.EcoKI to the DNA duplex was monitored by anisotropy. Upon binding of M.EcoKI to DNA, the DNA–protein complex tumbles more slowly, resulting in an increase in fluorescence anisotropy from the fluorophore on the DNA.
